# Contraction intensity modulates spinal excitability during transcranial magnetic stimulation-evoked silent period in rectus femoris muscle

**DOI:** 10.1007/s00421-023-05367-1

**Published:** 2023-11-30

**Authors:** Gonzalo Gomez-Guerrero, Paul Ansdell, Glyn Howatson, Janne Avela, Simon Walker

**Affiliations:** 1 NeuroMuscular Research Center (NMRC), Faculty of Sport and Health Sciences, University of Jyväskylä, Viveca (VIV221), 40700 Jyväskylä, Finland; 2 Faculty of Health and Life Science, Northumbria University, Newcastle Upon Tyne, UK; 3 Water Research Group, North West University, Potchefstroom, South Africa

**Keywords:** Lumbar stimulation, Spinal inhibition, Lower limbs, Force production, Cortico-spinal tract

## Abstract

**Purpose:**

Reduced spinal excitability during the transcranial magnetic stimulation (TMS) silent period (SP) has recently been shown to last longer than previously thought in the upper limbs, as assessed via spinal electrical stimulation. Further, there is reason to expect that contraction intensity affects the duration of the reduced spinal excitability.

**Methods:**

This study investigated spinal excitability at different time delays within the TMS-evoked SP in m.rectus femoris. Fifteen participants performed non-fatiguing isometric knee extensions at 25%, 50% and 75% of maximum voluntary contraction (MVC). Lumbar stimulation (LS) induced a lumbar-evoked potential (LEP) of 50% resting M-max. TMS stimulator output induced a SP lasting ~ 200 ms. In each contraction, a LEP (unconditioned) was delivered ~ 2–3 s prior to TMS, which was followed by a second LEP (conditioned) 60, 90, 120 or 150 ms into the silent period. Five contractions were performed at each contraction intensity and for each time delay in random order.

**Results:**

Compared to the unconditioned LEP, the conditioned LEP amplitude was reduced (− 28 ± 34%, *p* = 0.007) only at 60 ms during 25% of MVC. Conditioned LEP amplitudes during 50% and 75% of MVC were reduced at 60 ms (− 37 ± 47%, *p* = 0.009 and − 37 ± 42%, *p* = 0.005, respectively) and 150 ms (− 30% ± 37%, *p* = 0.0083 and − 37 ± 43%, *p* = 0.005, respectively). LEP amplitude at 90 ms during 50% of MVC also reduced (− 25 ± 35%, *p* = 0.013).

**Conclusion:**

Reduced spinal excitability is extended during 50% and 75% of MVC. In future, paired TMS-LS could be a potential method to understand changes in spinal excitability during SP (at different contraction intensities) when testing various neurophysiological phenomena.

## Introduction

Transcranial Magnetic Stimulation (TMS) applied over the contralateral motor cortex of the muscle targeted, in relaxed and active conditions, produces a muscle action potential that can be recorded by electromyography (EMG) and a muscle twitch. The muscle action potential is referred to as the motor-evoked potential (MEP) and provides information about cortico-spinal excitability (Barker et al. 1985; Day et al. 1989a). In addition, when TMS is applied during voluntary muscle contraction there is an interruption of the background EMG activity after the MEP ( Mills 1988; Day et al. 1989b). This interruption is known as the TMS-evoked silent period (SP) and its duration provides information about inhibition of the cortico-spinal tract (Inghilleri et al. 1993; Triggs et al. 1993; Taylor et al. 1996).

For some time, changes in the length of SP have been considered as an indicator of altered intracortical inhibition (Kidgell et al. 2013; Ruotsalainen et al. 2014; Manca et al. 2016; Latella et al. 2017). However, while reduced MEP amplitude, as an indicator of intracortical inhibition, has indeed been shown during the TMS-evoked SP, studies have consistently shown concomitant decreases in spinal excitability 50–100 ms after TMS that evokes a ~ 200 ms SP (Fuhr et al. 1991; Inghilleri et al. 1993; McDonnell et al. 2006; McNeil et al. 2009). Reduced spinal excitability is possibly due to motor-neuron afterhyperpolarization (AHP) and/or recurrent inhibition (RI) via Renshaw cells (RC), as well as Ia interneuron unloading through reciprocal inhibition (Mills 1988; Fuhr et al. 1991; Ziemann et al. 1993). Interestingly, a recent study showed reduced spinal excitability up to 150 ms in the upper limbs after TMS, which was argued to be attributed to an increase in Golgi tendon organ (GTO) activity and muscle spindle unloading (Yacyshyn et al. 2016). Thus, emerging evidence suggests that spinal excitability is modulated over a longer proportion of SP than previously thought.

One experimental consideration is that traditional H-reflex methodology used in previous studies (Fuhr et al. 1991; Ziemann et al. 1993) limits the assessment of modified spinal excitability < 100 ms, as the measure reflects modified pre-synaptic inhibition. In contrast, direct percutaneous activation of the spinal cord predominantly activates monosynaptic cortico-spinal tract axons (Taylor 2006; McNeil et al. 2013) and can be applied during both submaximal and maximal contractions (Petersen et al. 2002; Škarabot et al. 2019a). It would, therefore, be appropriate to test whether there is reduced spinal excitability at time delays greater than 100 ms (Yacyshyn et al. 2016) in the lower-limbs, since previous studies have relied on H-reflex methodology (Ziemann et al. 1993). While spinal responses can be elicited at cervical (cervicomedullary-evoked potential (CMEP)) and thoracic (thoracic motor-evoked potential (TMEP)) (Martin et al. 2008) segments of the spine, recent studies suggested that lumbar stimulation (lumbar-evoked potentials (LEP)) are a valid (Škarabot et al. 2019a) and more tolerable (Brownstein et al. 2020) method to study spinal excitability of the lower-limbs.

One final consideration is that contraction intensity could affect the duration of the reduced spinal excitability during the TMS-evoked SP. Increases in voluntary torque production increase the tension of the tendon and, consequently, increase GTO activity (Houk et al. 1970). In addition, muscle relaxation rate following TMS is greater with increased torque, which could activate muscle spindles as the sarcomeres lengthen (Vernillo et al. 2022). As such, afferent feedback mechanisms may be modified by increased torque level and potentially influence spinal excitability during SP. In the knee extensors, contractions of 25% of maximal voluntary contraction (MVC) resulted in the unconditioned TMEP being the same amplitude as the subsequent (TMS-) conditioned TMEP evoked at a time delay of 100 ms (Finn et al. 2018). In another study, the conditioned TMEP amplitude at a time delay of 100 ms was decreased when contracting to 50% of MVC (Brownstein et al. 2020). These results suggest contrasting responses between 25 and 50% of MVC.

Examining the contributing factors to the SP in locomotor muscles is important for determining exercise-induced alterations in nervous system function throughout the spectrum of health, exercise and disease (Sidhu et al. 2013). Consequently, there is a need to directly examine the duration of spinal inhibition within the TMS-evoked SP in the lower-limbs across different contraction intensities. The purpose of the study was to assess spinal excitability at different time delays (60, 90, 120 and 150 ms) within the TMS-evoked SP in the rectus femoris (RF) muscle with lumbar stimulation (LS) at different contraction intensities (25, 50, and 75% of MVC). It was hypothesized that reduced spinal excitability would be observed at longer time delays within the SP at increasing contraction intensities.

## Material and methods

### Participants

Twenty-two healthy adults (8 female) volunteered for the study. Seven participants were not considered due to possible activation of ventral roots (see Lumbar-evoked potentials). Therefore, the data presented here are representative of the 15 (4 female) volunteers fulfilling all study requirements (males: 11 subjects, 31 ± 6 years, height 178 ± 6 cm, weight 82 ± 8 kg; females: 4 subjects, 28 ± 1 years, height 166 ± 8 cm, weight 64 ± 7 kg). All included participants were free from neurological illness and musculoskeletal injury in the lower-limbs for the last 6 months, were not taking any medications known to affect the nervous system and had no contraindications to transcranial magnetic stimulation (TMS), which was assessed via a health questionnaire (modified from Rossi et al. (2009). Before testing, all participants were fully informed of the procedures and possible risks, and each participant provided written inform consent. The study was approved by the Ethical committee of the University of Jyväskylä (10.01.2020) and was conducted with accordance with the *Declaration of Helsinki* (2013).

An a priori sample size estimation was conducted using G*Power software (version 3.1, University of Dusseldorf, Germany), based on data presented by Yacyshyn et al. (2016) for *α* = 0.05 and power = 0.80. The estimated sample size needed was 18 participants to assess torque × time delay interaction between unconditioned and conditioned LEPs.

### Experimental set‑up

Detailed description of Torque, M-max, TMS, Lumbar stimulation and EMG can be found in the subsections below.

Participants visited the laboratory on one occasion. To assess responses in the RF muscle, participants were sat in a custom-built chair with a calibrated load cell (Faculty of Sport and Health Sciences, University of Jyväskylä, Finland) with hip and knee at 90° flexion and the shin strapped with a non-elastic restraint ~ 2 cm superior to the ankle malleoli. The voltage signal originating from the load cell was calibrated and converted into torque (N·m). All measures were performed on the right (i.e., dominant) leg, assessed by self-report of which foot they primarily kick a ball (van Melick et al. 2017).

Once the participant was secured to the dynamometer, the maximum compound action potential (M-max) was assessed in a relaxed condition. Two maximal voluntary contraction (MVC) trials were performed 60 s apart. Prior to the MVC, two contractions at ~ 50% and ~ 80% of estimated MVC were performed as a warm-up. Verbal encouragement and visual feedback were provided to motivate participants to produce maximal effort. Thereafter, target contraction intensities (25%, 50% and 75% of MVC) were displayed on the screen as visual feedback for the participant.

Placement of the lumbar stimulation electrodes was assessed to avoid activating spinal nerve roots (see Lumbar-evoked potentials). Thereafter, stimulator intensity was adjusted to produce a LEP of 50% of the M-max at rest, and this stimulation intensity was used throughout the experiment. TMS coil placement was defined as the location producing the largest MEP in the RF, and stimulator output intensity was standardized to evoke ~ 200 ms SP from the stimulator artefact to the resumption of the voluntary EMG signal, during brief voluntary contractions at each torque.

**Fig. 1:**
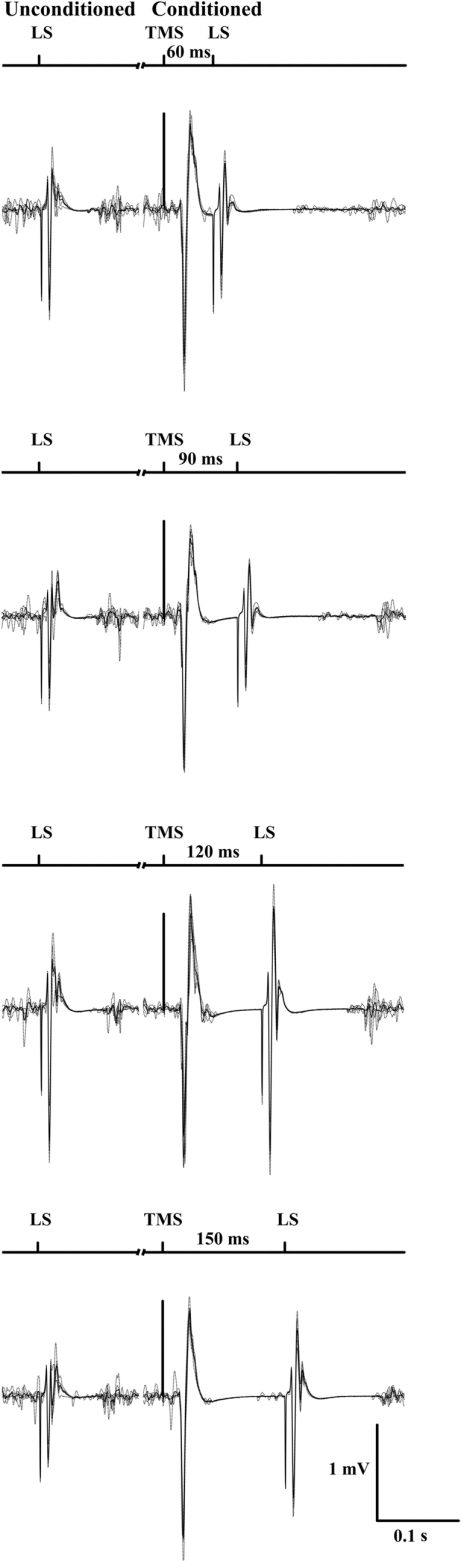
One participant’s mean (solid) and individual (dashed) trials that represent the experimental design of one set of unconditioned and conditioned lumbar stimulation at different time delays taken from 25% MVC trials. *TMS* transcranial magnetic stimulation, *LS* lumbar stimulation

During the session, unconditioned and conditioned LEPs were delivered during the same voluntary contraction. Unconditioned LEP consisted of a single stimulation delivered at the lumbar level. Conditioned LEPs consisted of a paired stimulation of TMS followed by lumbar stimulation separated by predetermined and randomly ordered time delays (60, 90, 120 and 150 ms). Participants were instructed to contract to, and briefly hold, one of the three different contraction intensities (25, 50 and 75% of MVC) in a randomized order. Once the participant reached the required level, an unconditioned LEP was delivered followed by a conditioned LEP at one of the different time delays (Fig. 1). The contractions were held for 5–8 s and stimuli were delivered 2–3 s apart. Sets of five unconditioned, followed by conditioned LEPs, were given per time delay and per torque level as a single block, giving a total of 60 unconditioned and conditioned stimuli. To avoid fatigue (see Results), 30, 45 and 60 s rest was given between contractions at 25%, 50% and 75% of MVC, respectively, and 60, 120 and 180 s rest was given between the sets of 5 contractions. At the end of the protocol, M-max and MVC were reassessed.

### Peripheral nerve stimulation

Percutaneous electrical stimulation of the femoral nerve (3.2 cm cathode/anode arrangement; Polar Neurostimulation Electrodes, Espoo, Finland) was performed to elicit M-max in RF (1 ms square pulse duration; Digitimer DS7AH, Hertfordshire, UK). Electrodes were placed 2 cm apart and placed at each side of the femoral nerve, located by palpation and identification of the femoral artery (Walker et al. 2016). M-max was elicited by gradually increasing stimulator output intensity until the EMG response plateaued. To ensure supramaximality, this intensity was further increased by 50% (mean ± standard deviation intensity: 257 ± 151 mA).

### Transcranial magnetic stimulation

Single TMS pulses were delivered using a Magstim 200^2^ magnetic stimulator (Magstim Co., Ltd., Whitland, UK) connected to a concave double-cone coil, positioned over the left cortical hemisphere for RF with a posterior-to-anterior current orientation. The hotspot was defined, at rest, as the position eliciting the largest MEP recorded in the EMG using the same intensity (i.e., 50–70% stimulator output) producing a visible MEP. The coil position was marked on the scalp, once the hotspot was found, to maintain the same position throughout the protocol. Stimulus intensities were set to evoke a silent period of ~ 200 ms for all contraction intensities (Table 1).

**Table 1:** Mean and standard deviation values of MEP, lumbar stimulation and involuntary EMG activity parameters from the participants at different submaximal torque levels

	25% MVC	50% MVC	75% MVC
TMS stimulator output (%)	66 ± 16	64 ± 12	65 ± 14
MEP SP: SORE (ms)	216 ± 15	210 ± 10	216 ± 14
MEP (mV)	2.16 ± 1.35	2.02 ± 1.10	1.79 ± 0.84
LEP latency (ms)	6.3 ± 0.7	6.6 ± 0.7	6.6 ± 0.5
Involuntary EMG activity amplitude (mV)	0.11 ± 0.07	0.14 ± 0.09	0.20 ± 0.14

These values represent the standardization of the measurement

*MVC* maximal voluntary contraction, *TMS* transcranial magnetic stimulation, *MEP* motor evoked potential, *SP* silent period, *SORE* stimulation offset to return of electromyography, *LEP* lumbar evoked potential

### Lumbar-evoked potentials

LEPs were elicited with a constant-current stimulator (1 ms square pulse duration; Digitimer DS7AH, Hertfordshire, UK) via self-adhesive electrodes (Polar Neurostimulation Electrodes, Espoo, Finland). The cathode (5 × 10 cm) was centered over the first lumbar vertebra (L_1_) and the anode (circular shape; 3.2 cm diameter) was placed on the midline of the vertebral column ~ 5 cm above the top edge of the cathode as described by Škarabot et al. (2019a).

The intensity of stimulation (309 ± 108 mA) was standardized to 50% of the M-max evoked in the resting position. Potential activation of ventral roots was assessed by examining the onset latency of the LEP with an increase in stimulator intensity (Petersen et al. 2002) and tracking LEP amplitude during increased voluntary contraction (Taylor et al. 2002). Should the ventral roots be activated by the stimulation procedures, onset latency would have shortened with an increase in stimulator intensity and LEP amplitude would have been the same during increased voluntary contraction (Petersen et al. 2002; Taylor et al. 2002, 2006; Škarabot et al. 2019a).

**Fig. 2:**
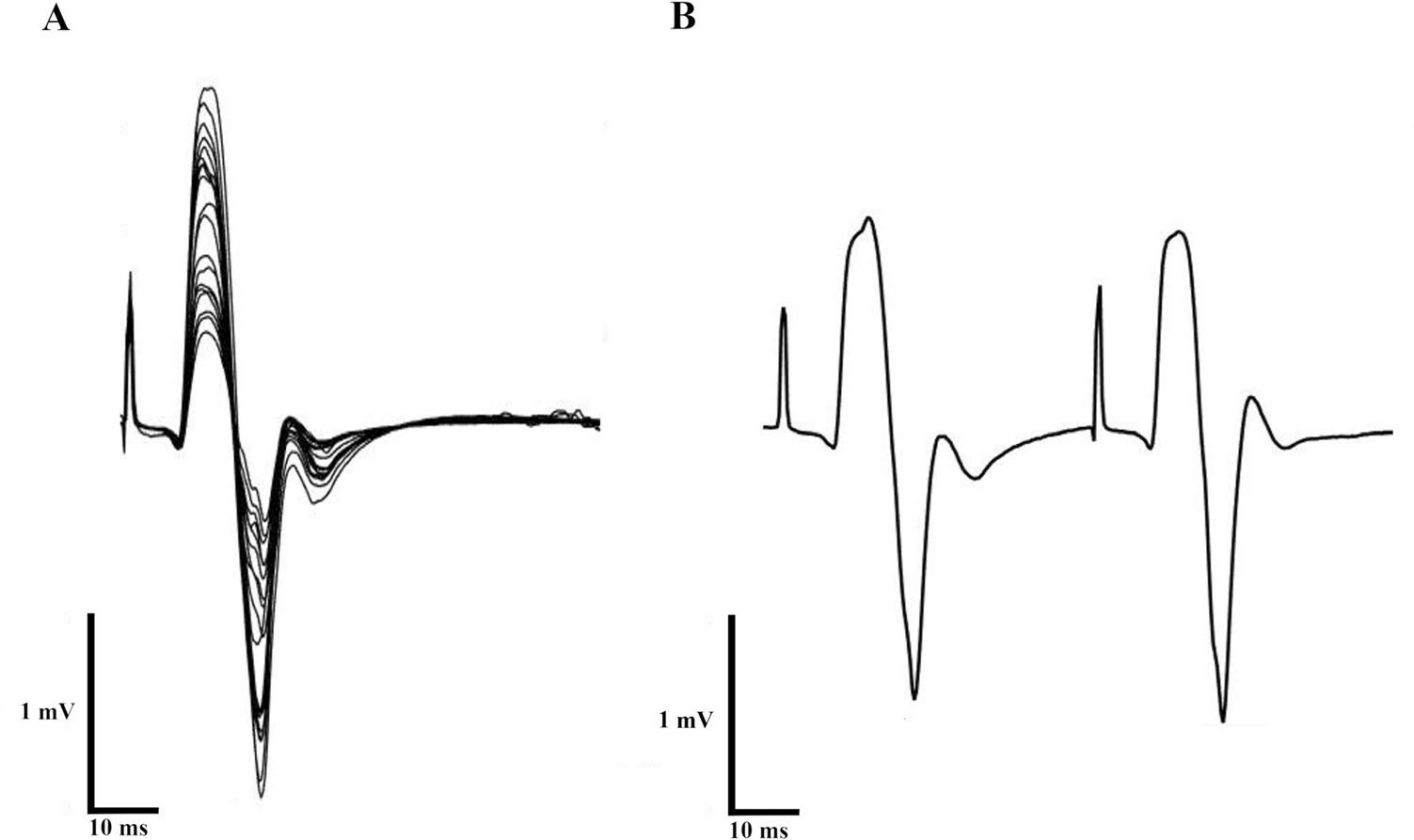
Data extracted from one participant showing that spinal root activation did not occur. **A** When increasing the intensity of stimulator output there was no reduction in latency. **B** A lumbar stimulated doublet with 50 ms interval, showing similar amplitudes between the stimulations

Dorsal root activation was assessed via paired LS with 50 ms time delay (Fig. 2), where the amplitude of the second LEP was compared to the first. Evidence of dorsal root activation would be a decrease in the second LEP due to post-activation depression at the motor-neuron pool (Hofstoetter et al. 2018). All remaining participants showed no sign of the responses described and reported that they found LS to be tolerable.

### Bipolar surface electromyography and torque

Muscle activity was recorded using adhesive Ag/AgCl electrodes (3 × 2 cm, BlueSensor N, Ambu, Penang, Malaysia) from m.Bicep Femoris (BF) and RF according to SENIAM Guidelines (Hermens et al. 2000). Skin was shaved, abraded with sandpaper, and wiped with alcohol before setting the electrodes in bipolar arrangement with 2 cm center-to-center distance. Impedance was set < 2kΩ, and the reference electrode was positioned above the patella. EMG data were amplified (1000 ×), bandpass filtered (16–1000 Hz; Neurolog System, Digitimer Ltd, UK)) and sampled online at 3000 Hz using CED Power1401-3 (Cambridge Electronic Design Ltd, Cambridge, UK).

Torque was sampled at 1000 Hz, amplified by a custom-built amplifier (ForAmps 1 v1.2, University of Jyväskylä, Finland) and converted by a 16-bit A/D board (CED Power1401-3, Cambridge Electronics Design, Cambridge, UK) in combination with Spike2 software (version 6.10, Cambridge Electronic Design, Cambridge, UK).

### Data and statistical analyses

Offline analyses were performed with Spike software (version 6.10, Cambridge Electronic Design, Cambridge, UK) to manually obtain M-max amplitude, MVC, MEP Silent Period and unconditioned LEP onset latencies. The other outcome measures were analyzed by a customized MATLAB script (version R2020b, The MathWorks, Inc., Natick, USA). Peak-to-peak amplitude of LEPs and MEPs was analyzed automatically between latencies-of-interest following peripheral nerve stimulation, lumbar stimulation or TMS (Taylor et al. 1999), respectively. Torque was averaged over the 100 ms before the stimulator artefact. SP duration was determined, through visual inspection, as the time from the stimulator artefact to the return of voluntary EMG (Damron et al. 2008).

**Fig. 3:**
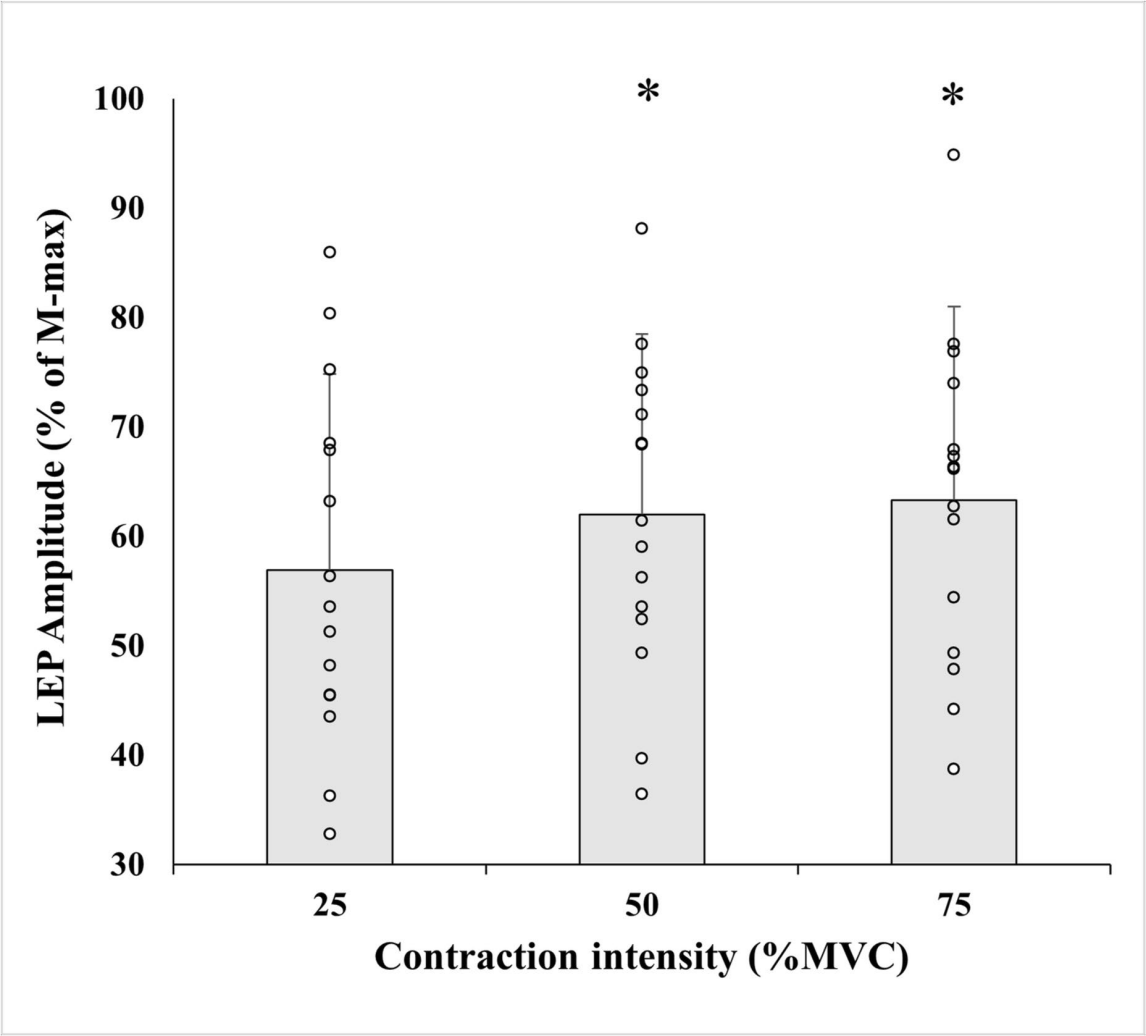
Mean (± SD) and individual values of unconditioned LEP response normalized to M-max at different contraction intensities. Increases in LEP amplitude with increases in torque shows that the stimulation was evoked trans-synaptically

SPSS software (version 26.0, SPSS Inc., Chicago, USA) was used for all statistical methods. Means and standard deviation (SD) were calculated and reported throughout. Normality of the data was tested with the Shapiro–Wilk test and confirmed by z-score with an acceptance of + 2 to -2 (e.g. skewness score/skewness score_SE_ and kurtosis score/kurtosis score_SE_). Data that did not fulfil those requirements were Log10 transformed, which then fulfilled the requirements for Normality. Paired t-tests were used to assess possible effects of fatigue between M-maxpre and M-maxpost, MVCpre and MVCpost, and to evaluate unconditioned LEP amplitude at different torque levels in the control measurements (shown in Fig. 3). One-way analysis of variance (ANOVA) was used to assess potential differences between the three contraction intensities in control measures: Unconditioned LEP latencies, MEP amplitude and MEP Silent Period (shown in Table 1). To determine whether Normalized [Conditioned/Unconditioned LEP*100] LEPs responded differently at the tested time delays between the three different torque levels, two-way repeated measures ANOVA was employed. When sphericity assumptions were violated, Greenhouse–Geisser corrections were used. Post-hoc Bonferroni adjustments were used when significant main effects were found. When comparing Unconditioned and Conditioned LEP at each time delay, the Benjamin–Hochberg test corrected for multiple paired *t* test comparisons with a 10% false discovery rate. Effect sizes are represented as partial eta-squared values (*η*_*p*_^*2*^ = small: 0.01, medium: 0.06, large: 0.14) for the factors of the ANOVA and as Hedge’s *g* for between-group effect sizes for these relative changes (*g* = small: < 0.3, medium: 0.3–0.8, large: > 0.8). Αlpha was set at 0.05.

## Results

### Control measurements

There were no statistically significant differences between time delays for MEP amplitude during 25% of MVC (*F*_(3, 56)_ = 0.033, *p* = 0.992), during 50% of MVC (*F*_(3, 56)_ = 0.024, *p* = 0.995), or during 75% of MVC (*F*_(3, 56)_ = 0.191, *p* = 0.902). Additionally, there were no statistical differences between SP duration at any contraction intensity (*F*_(2 42)_ = 1.110, *p* = 0.339), indicating standardized conditions throughout the experiment to examine spinal excitability.

There were no statistically significant differences between M-maxpre and M-maxpost (M-maxpre = 3.27 ± 1.13 mV, M-maxpost = 2.96 ± 1.04 mV, *p* = 0.054, 95% CI [− 0.01, 0.62], Hedges’ *g* = 0.27) nor between MVCpre and MVCpost (MVCpre = 221 ± 60 N·m; MVCpost = 214 ± 54 N·m, *p* = 0.106, 95% CI [− 1.74, 15.25], Hedges’ *g* = 0.12).

LEP latencies did not show statistical difference between time delays during 25% of MVC (*F*_(3, 56)_ = 0.106, *p* = 0.956), during 50% of MVC (*F*_(3, 56)_ = 0.016, *p* = 0.997) or during 75% of MVC (*F*_(3, 56)_ = 0.153, *p* = 0.902). There was a statistically significant difference between unconditioned LEP amplitude during 25% vs 50% of MVC (*p* < 0.001, 95% CI [− 1.74, 15.25], Hedges’ *g* = − 0.26) and 25% vs 75% (*p* = 0.001, 95% CI [− 0.21, − 0.06], Hedges’ *g* = − 0.27) of MVC, although no statistical difference was found between 50% of MVC and 75% of MVC (*p* = 0.956, 95% CI [− 0.05, 0.05], Hedges’ *g* = − 0.01) (Fig. 3). Collectively, these findings indicate that LS activated the cortico-spinal tract.

### Effects of torque on spinal excitability at different time delays

**Fig. 4:**
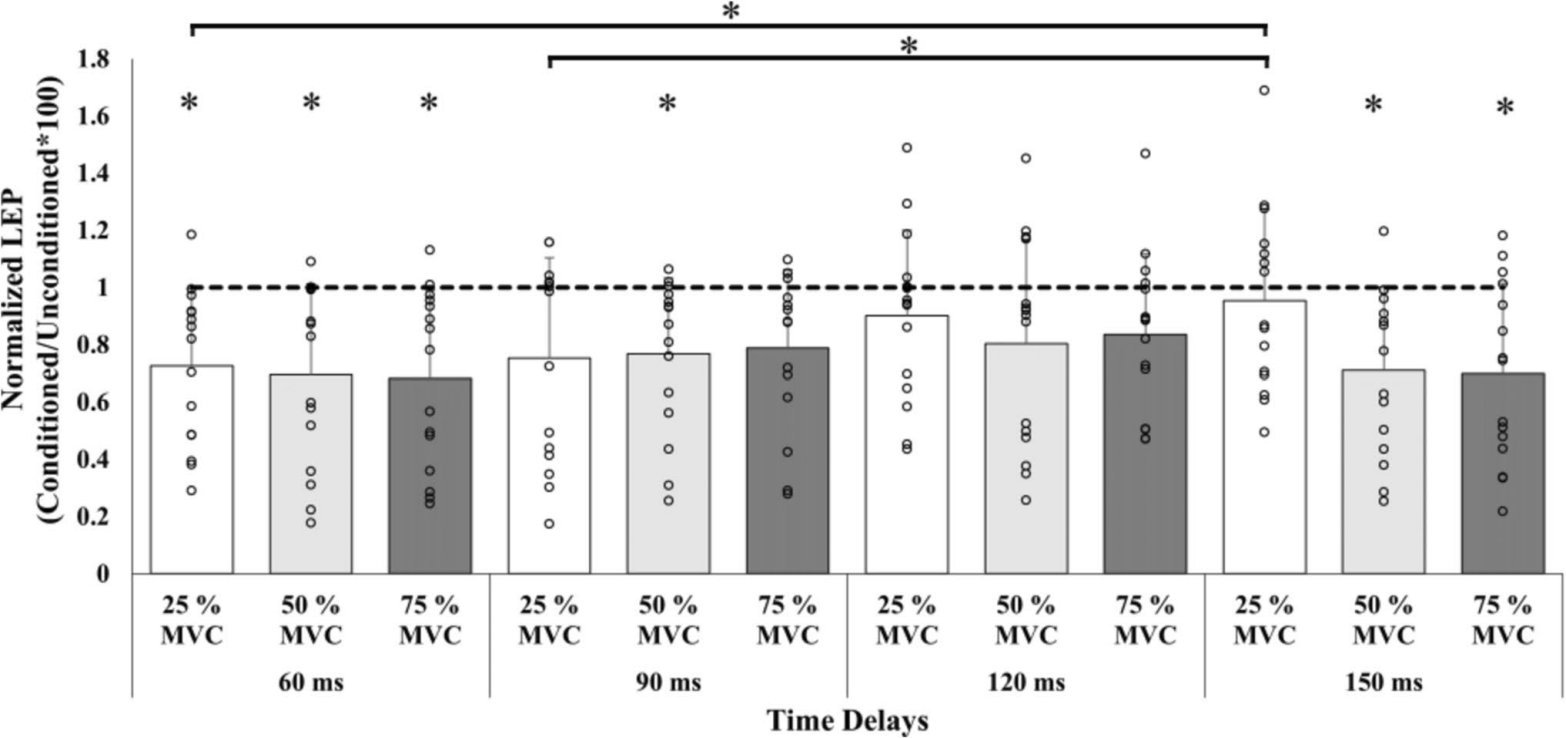
Mean (± SD) and individual values of conditioned LEP normalized to the unconditioned LEP. The dashed line represents the unconditioned LEP amplitude. Any data point or bar below the dashed line represents inhibition and any data or bar above the dashed line represents facilitation of the conditioned LEP. Bars represent the mean values at each contraction intensity and time delay. The circles represent each participant’s data at each contraction intensity and time delay. *p < 0.05 vs unconditioned LEP amplitude

Two-way repeated measures ANOVA showed a significant main effect between time delays (*F*_(2,5, 102.4)_ = 6.542, *p* = 0.001, *η*_*p*_^*2*^ = 0.135) and torque × time delay interaction (*F*_(4.9, 102.4)_ = 2.953, *p* = 0.016, *η*_*p*_^*2*^ = 0.123) for the normalized LEP. Post hoc analyses revealed significant difference in LEP amplitude between 60 ms (0.73 ± 0.27) and 150 ms (0.95 ± 0.34) (*p* = 0.007, 95% CI [− 0.398, − 0.046], Hedges’ *g* = − 0.27) and 90 ms (0.75 ± 0.35) and 150 ms (*p* = 0.004, 95% CI [− 0.352, − 0.050], Hedges’ *g* = − 0.25) during 25% of MVC (Fig. 4).

### Unconditioned vs conditioned LEP

Unconditioned LEP was compared to the conditioned LEP at each time delay at the three contraction intensities. During 25% of MVC, conditioned LEP amplitude was statistically lower than unconditioned LEP at 60 ms (*t*_(14)_ = − 3.128, *p* = 0.007, 95% CI [− 0.464, − 0.087], Hedges’ *g* = − 0.62), but not at 90 ms (*t*_(14)_ = − 2.397, *p* = 0.075, 95% CI [− 0.505, − 0.028], Hedges’ *g* = − 0.58), 120 ms (*t*_(14)_ = − 1.285, *p* = 0.220, 95% CI [− 0.292, 0.073], Hedges’ *g* = − 0.18), nor 150 ms (*t*_(14)_ = 0.722, *p* = 0.482, 95% CI [− 0.248, 0.123], Hedges’ *g* = − 0.13).

During 50% of MVC, statistical differences were found at 60, 90 and 150 ms (*t*_(14)_ = − 3.052, *p* = 0.009, 95% CI [− 0.634, − 0.111], Hedges’ *g* = − 0.76, *t*_(14)_ =  − 2.843, *p* = 0.013, 95% CI [− 0.446, − 0.062], Hedges’ *g* = − 0.44 and *t*_(14)_ = − 3.099, *p* = 0.008, 95% CI [− 0.502, − 0.091], Hedges’ *g* = − 0.52, respectively), where the conditioned LEP was lower than the unconditioned LEP. There were no statistically significant differences in conditioned versus unconditioned LEP amplitude at 120 ms (*t*_(14)_ = − 2.073, *p* = 0.057, 95% CI [− 0.451, 0.008], Hedges’ *g* = − 0.36).

During 75% of MVC, the conditioned LEP amplitude was significantly lower than unconditioned LEP (Fig. 4) at 60 ms and 150 ms (*t*_(14)_ = − 3.348, *p* = 0.005, 95% CI [− 0.602, − 0.132], Hedges’ *g* = − 0.78, and *t*_(14)_ = − 3.377, *p* = 0.005, 95% CI [− 0.610, − 0.136], Hedges’ *g* = − 0.70, respectively). But no statistically significant differences were observed at 90 ms nor 120 ms (*t*_(14)_ = − 2.511, *p* = 0.067, 95% CI [− 0.429, − 0.034], Hedges’ *g* = − 0.51 and *t*_(14)_ = − 2.626, *p* = 0.083 (corrected), 95% CI [− 0.394, − 0.040], Hedges’ *g* = − 0.52, respectively).

## Discussion

This is the first study to directly test spinal excitability at different time delays during TMS-evoked SP, and during different contraction intensities, in the lower-limbs (specifically RF). Our results showed reduced spinal excitability during the first 60 ms in RF during all contraction intensities, extending to 90 ms at 50% of MVC and further reductions were observed at 150 ms during 50 and 75% of MVC.

These results conflict with a previous study that used CMEPs during a 25% of MVC contraction in upper limb (Yacyshyn et al. 2016); the conditioned CMEP showed differences from the unconditioned response also at 120 and 150 ms after TMS. However, our results agree with early studies conducted using H-reflex methodology in both upper- and lower-limbs (Fuhr et al. 1991; Ziemann et al. 1996) despite that H-reflex data could be influenced by changes in presynaptic inhibition, which is absent in our methods. The results suggest that reduced spinal excitability is present but largely limited to ≤ 90 ms after TMS in lower-limb muscles, at low contraction intensities (i.e., < 25%of MVC). Nevertheless, differences between upper- and lower-limbs have previously been presented by Giesebrecht et al. (2010). They reported a facilitatory response to spinal stimulation in tibialis anterior after 10 s MVC, in contrast of spinal inhibition observed by Gandevia et al. (1999) in biceps brachii after 5–10 s MVC contraction, discussing different physiological mechanisms in upper- and lower-limbs muscles.

Compiling the existing literature provides indirect support for the present study’s finding in that contraction intensity influenced the duration of reduced spinal excitability during SP. First, Finn et al. (2018) did not observe reduced spinal excitability at 100 ms (TMS induced a 200 ms SP), given that the conditioned TMEP was similar to the amplitude of the unconditioned TMEP when standardized to 50% of the M-max (as in the current study). Conversely Brownstein et al. (2021) did observe reduced spinal excitability since both conditioned TMEP and LEP amplitude at 100 ms (TMS included 200 ms SP) were lower than their respective unconditioned amplitudes, again when spinal stimulation was standardized at 50% of the M-max. As Finn et al. (2018) employed contraction intensities of 25% of MVC, whereas Brownstein et al. (2021) employed 50% of MVC, this suggests that contraction intensity influences the duration of reduced spinal excitability. In directly assessing this hypothesis, spinal excitability was reduced at 60 ms but no longer at 90 ms after TMS contracting to 25% of MVC, matching the findings of Finn et al. (2018). However, reductions in conditioned LEP were observed at 90 ms during 50% of MVC and at 150 ms during 50% and 75% of MVC, providing support for and extending the findings of Brownstein et al. (2021). Thus, we suggest that increased contraction intensity modulates spinal excitability distinctly in that reduced stimulation-induced responses are apparent at longer time delays when contracting at a higher intensity.

The suggested mechanisms for the decrease in spinal excitability during TMS-evoked SP are: afterhyperpolarization (AHP), recurrent inhibition via Renshaw cells, Ia interneuron unloading through reciprocal inhibition, and/or GTO inhibition (Mills 1988; Fuhr et al. 1991; Ziemann et al. 1993; Yacyshyn et al. 2016). Although AHP, RI and GTO inhibition are dependent on the preceding motor-neuron activity (Hultborn & Pierrot-Deseilligny 1979; Ziemann et al. 1993) and the size of the conditioned test stimuli (Hultborn & Pierrot-Deseilligny 1979), AHP may not account for more than ~ 56 ms, since discharge rate at 50% of MVC is ~ 18 pps in the VL (Kamen & Knight 2004). There is evidence that AHP could impact excitability up to approx. 100 ms, depending on motor-neuron firing rate (Piotrkiewicz et al. 2007), as observed in upper-limb muscles. Thus, the exact duration of the influence of AHP is still unresolved in different muscles. However, converging evidence suggests that this may not be the case in explaining the difference between conditioned LEP amplitude during 25% versus 50% of MVC at 90 ms in the present study.

Among the TMS-evoked SP studies, Ziemann et al. (1993) found that the conditioned/unconditioned H-reflex amplitude progressively decreased with increasing contraction intensity in the soleus muscle (SOL). The authors argued that Renshaw cells might have a stronger influence on TMS-evoked SP inhibition, rather than GTOs or muscle spindles, since the decrease in spinal excitability was ~ 50 ms, and those monosynaptic feedback mechanisms start to exert an influence after ~ 40 ms in SOL. Although RI may only account for ~ 40 ms (Pierrot-Deseilligny & Burke 2005), it could influence discharging rate (Granit et al. 1960). Since stimulator output was not statistically different in 25% and 50% of MVC conditions, a plausible mechanism to explain the prolonged decrease from 60 to 90 ms in spinal excitability at higher contraction intensities could be recurrent inhibition via Renshaw cells.

In the present study, the interstimulus intervals of 60 and 90 ms could also be affected by modified muscle spindle or GTO activity to the cortico-spinal tract. The spindles provide muscle length feedback and GTOs provide tensile feedback (Enoka 2008; Nichols 2018). When there is an increase in contraction intensity, GTOs increase their discharge rate, increasing Ib inhibition (Houk et al. 1970). Further, the TMS-induced muscle twitch has been suggested to also engage GTOs increasing Ib inhibition (Yacyshyn et al. 2016). It is conceivable that the combination of higher intensity contractions and muscle twitch-induced Ib inhibition could be enhanced in the present study’s 50% of MVC trials. Therefore, GTOs may be one candidate for the continued decrease of spinal excitability with increasing contraction intensity.

**Fig. 5:**
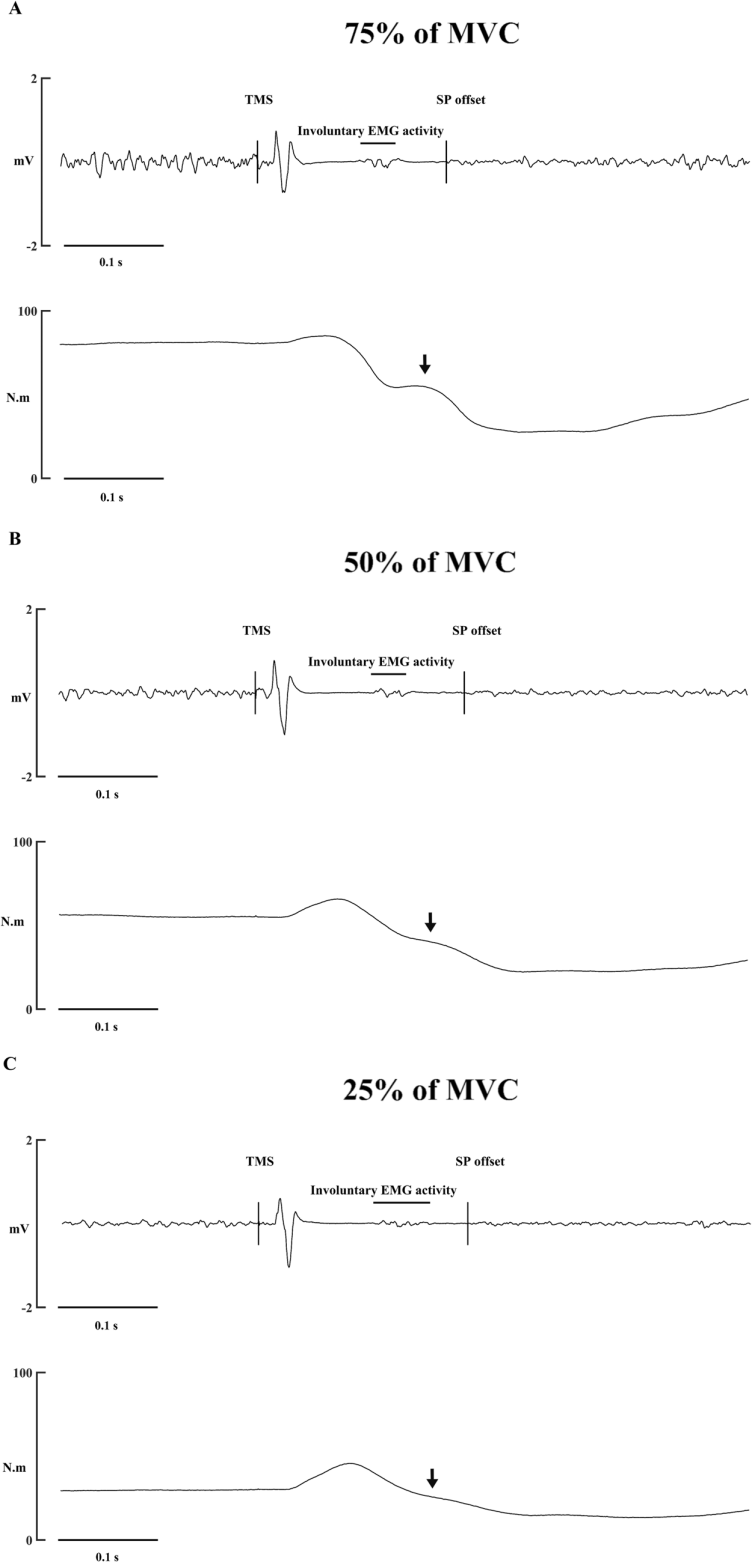
Involuntary EMG activity during the SP of a participant during different trials at **A** 75% of MVC, **B** 50% of MVC and **C** 25% of MVC. Upper traces represent the EMG signal and lower traces represent torque signal. The arrow points to the possible effect of the involuntary EMG in the torque trace. This phenomenon was observed in 11/15 participants. *TMS* transcranial magnetic stimulation, *SP* silent period

One interesting finding in the present study was the observed return of conditioned/unconditioned LEP to baseline during 25% and 75% of MVC at 90 ms and at 120 ms for all conditions, but then a second reduction in spinal excitability at 150 ms during 50% and 75% of MVC (Figs. 4 and 5). An involuntary EMG activity burst (80–150 ms) has been previously observed in upper- (Calancie et al. 1987; Holmgren et al. 1990; Butler et al. 2012) and lower-limbs (Dimitrijević et al. 1992), categorized as “low level EMG” (Butler et al. 2012) or “breakthrough EMG” (Hupfeld et al. 2020), and its origin is not known. But this involuntary EMG activity has been postulated to arise from cortical pathways (Holmgren et al. 1990; Dimitrijević et al. 1992), spinal reflex (Dimitrijević et al. 1992; Butler et al. 2012) and/or agonist and antagonist muscle activity, through polysynaptic excitatory and inhibitory potentials to the motor-neuron (Calancie et al. 1987). This involuntary activity was also observed in 11 of our 15 participants (Fig. 5), with onset latencies between 83 and 130 ms and lengths of 28–91 ms. Additionally, the size of the response increased at 75% vs 25% of MVC (Table 1). Muscle spindles have been considered as a mechanism for the involuntary EMG activity. After the TMS-evoked twitch, there is a period of relaxation, where sarcomeres lengthen and the muscle spindles could induce a monosynaptic reflex (Hupfeld et al. 2020; Škarabot et al. 2019b). Since increases in voluntary contraction increased the relaxation ratio and reduced the time to peak relaxation in knee extensor (Vernillo et al. 2022) muscle spindles could be responsible for the involuntary EMG activity. However, latencies of the patellar tendon reflex in RF were 16–22 ms (Frijns et al. 1997), and time to peak relaxation in knee extensors were ~ 140 ms and ~ 160 ms during contractions of 75% and 50% of MVC, respectively (Vernillo et al 2022). Thus, muscle spindles could provide feedback but not as early as the involuntary EMG activity observed in the present study. Consequently, one possible explanation for the return to baseline in spinal excitability at 90 ms during 75% of MVC and 120 ms during contractions > 50% of MVC could be afferent feedback provided by synergist and/or antagonist muscles from the same limb and contralateral limb (i.e., heteronymous feedback) (Houk et al. 1970; Calancie et al. 1987; Zehr et al. 2001; Wilmink & Nichols 2003; Manning & Bawa 2011). Wilmink & Nichols (2003) found that there were both excitatory and inhibitory effects from the vastii muscles on RF following stretches in cat forelimb. Furthermore, Zehr et al. (2001) showed a long-latency reflex in various muscles of the contralateral limb at 90 ms after peroneal nerve stimulation. Thus, at higher contraction intensities, heteronymous afferent signalling could be responsible for the return of spinal excitability at 90–120 ms, via an excitatory reflex that alters motor-neuron excitability at such time delays. Thus, we speculate that heteronymous feedback specifically affected the 120 ms time delay (and to a certain extent also the 90 ms delay) no longer influences conditioned LEP amplitude at 150 ms, allowing reduced spinal excitability to be observed with the lumbar stimulation method at higher contraction intensities. Nevertheless, this proposal should be specifically investigated in future.

### Strength and limitations

A strength of the study is the use of LS methodology to assess spinal excitability of the lower-limbs, which targets the cortico-spinal tract directly, and the positioning of the electrodes has been verified via response tests. These procedures are in-line with those of Škarabot et al. (2019a) who showed that LS can activate the cortico-spinal tract without activating dorsal and ventral roots.

Nevertheless, limitations need to be considered in the present study. TMS during different trials were not employed, in addition to spinal electrical stimulation, to compare cortico-spinal and spinal excitability at the same time delays (60, 90, 120 and 150 ms). This could have provided information regarding ongoing cortical inhibition along with spinal level inhibition (as employed by Fuhr et al. (1991) and Inghilleri et al. (1993). However, the number of trials needed would have compromised the present study’s ability to restrict neuromuscular fatigue during the testing session and tripled the number of transcranial stimulations. Second, we acknowledge that employing voluntary contractions in the present study’s methodology does not allow controlling for the background EMG activity/torque (Škarabot et al. 2019b) when unconditioned and conditioned LEP were elicited, since the unconditioned LEP was elicited during a period of voluntary muscle activity as opposed to during the SP. Third, sample size estimation suggested that 18 participants were needed to obtain medium effect sizes for torque × time delay interaction. We observed a significant interaction in normalized LEP but post-hoc comparisons have likely been underpowered to detect pairwise comparisons as only 15 participants were available for the final analysis.

## Conclusion

The present study confirmed that spinal excitability decreases up to 60 ms during the TMS-evoked SP in the lower-limbs when assessed through LS regardless of contraction intensity. Contraction intensity appeared to affect the duration of decreased spinal excitability, with evidence of reduced excitability at 150 ms during 50% and 75% of MVC and also reduced spinal excitability at 90 ms during 50% of MVC. Thus, interpretation of (changes in) SP duration being attributable to intracortical inhibition should be made with caution in future studies, particularly during higher contraction intensities. The present study demonstrates that paired TMS-LS could be a potential method to understand changes in spinal excitability (during SP at different contraction intensities) when testing various neurophysiological phenomena; e.g., examining acute fatigue or long-term adaptation.

## Data Availability

The datasets generated during and/or analyzed during the current study are available from the corresponding author on reasonable request.

## Abbreviations

AHP: Afterhyperpolarization
ANOVA: Analysis of variance
BF: Bicep femoris
CMEP: Cervicomedullary-evoked potential
EMG: Electromyography
GTO: Golgi tendon organ
H-reflex: Hoffmann’s reflex
kΩ: Kiloohm
L_1_: First lumbar vertebra
LEP: Lumbar-evoked potential
LS: Lumbar stimulation
MEP: Motor-evoked potential
M-max: Maximum compound action potential
Ms: Milliseconds
MVC: Maximal voluntary contraction
RC: Renshaw cells
RF: Rectus femoris
RI: Recurrent inhibition
s: Seconds
SOL: Soleus muscle
SORE: Stimulation offset to return of electromyography
SP: Silent period
TMEP: Thoracic motor-evoked potential
TMS: Transcranial magnetic stimulation

## References

[CR1] Barker AT, Jalinous R, Freeston IL (1985). Non-invasive magnetic stimulation of human motor cortex. The Lancet.

[CR2] Brownstein CG, Souron R, Royer N, Singh B, Lapole T, Millet GY (2020). Disparate kinetics of change in responses to electrical stimulation at the thoracic and lumbar level during fatiguing isometric knee extension. J Appl Physiol.

[CR3] Brownstein CG, Espeit L, Royer N, Ansdell P, Škarabot J, Souron R, Lapole T, Millet GY (2021). Reductions in motoneuron excitability during sustained isometric contractions are dependent on stimulus and contraction intensity. J Neurophysiol.

[CR4] Butler JE, Petersen NC, Herbert RD, Gandevia SC, Taylor JL (2012). Origin of the low-level EMG during the silent period following transcranial magnetic stimulation. Clin Neurophysiol.

[CR5] Calancie B, Nordin M, Wallin U, Hagabarth KE (1987). Motor-unit responses in human wrist flexor and extensor muscles to transcranial cortical stimuli. J Neurophysiol.

[CR6] Damron LA, Dearth DJ, Hoffman RL, Clark BC (2008). Quantification of the corticospinal silent period evoked via transcranial magnetic stimulation. J Neurosci Methods.

[CR7] Day BL, Dressler D, Maertens de Noordhout A, Marsden CD, Nakashima K, Rothwell JC, Thompson PD (1989). Electric and magnetic stimulation of human motor cortex: surface EMG and single motor unit responses. J Physiol.

[CR8] Day BL, Rothwell JC, Thompson PD, Maertens de Noordhout A, Nakashima K, Shannon K, Marsden CD (1989). Delay in the execution of voluntary movement by eletrical or magnetic brain stimulation in intact man. Brain.

[CR9] Dimitrijević MR, Kofler M, McKay WB, Sherwood AM, Van der Linden C, Lissens MA (1992). Early and late lower limb motor evoked potentials elicited by transcranial magnetic motor cortex stimulation. Electroencephalogr Clin Neurophysiol.

[CR10] Enoka RM (2008) Neuromechanics of human movement, 4th edn. Human Kinetics, pp 251–255

[CR11] Finn HT, Rouffet DM, Kennedy DS, Green S, Taylor JL (2018). Motoneuron excitability of the quadriceps decreases during a fatiguing submaximal isometric contraction. J Appl Physiol.

[CR12] Frijns CJM, Laman DM, Van Duijn MAJ, Van Duijn H (1997). Normal values of patellar and ankle tendon reflex latencies. Clin Neurol Neurosurg.

[CR13] Fuhr P, Agostino R, Hallett M (1991). Spinal motor neuron excitability during the silent period after cortical stimulation. Electroencephalogr Clin Neurophysiol.

[CR14] Gandevia SC, Petersen N, Butler JE, Taylor JL (1999). Impaired response of human motoneurones to corticospinal stimulation after voluntary exercise. J Physiol.

[CR15] Giesebrecht S, Martin PG, Gandevia SC, Taylor JL (2010). Facilitation and inhibition of tibialis anterior responses to corticospinal stimulation after maximal voluntary contractions. J Neurophysiol.

[CR16] Granit R, Haase J, Rutledget LT (1960). Recurrent inhibition in relation to frequency of firing and limitation of discharge rate of extensor motorneurons. J Physiol.

[CR17] Hermens HJ, Freriks B, Disselhorst-Klug C, Rau G (2000). Develompent of recommendations for SEMG sensors and sensor placement procedures. J Electromyogr Kinesiol.

[CR18] Hofstoetter US, Freundl B, Binder H, Minassian K (2018). Common neural structures activated by epidural and transcutaneous lumbar spinal cord stimulation: elicitation of posterior root-muscle reflexes. PLoS ONE.

[CR19] Holmgren H, Larsson LE, Pedersen S (1990). Late muscular responses to transcranial cortical stimulation in man. Electroencephalogr Clin Neurophysiol.

[CR20] Houk JC, Singer JJ, Goldman MR (1970). An evaluation of length and force feedback to soleus muscles of decerebrate cats. J Neurophysiol.

[CR21] Hultborn H, Pierrot-Deseilligny E (1979). Changes in Recurrent Inhibition during voluntary soleus contractions in man studied by an H-reflex technique. J Physiol.

[CR22] Hupfeld KE, Swanson CW, Fling BW, Seidler RD (2020). TMS-induced silent periods: a review of methods and call for consistency. J Neurosci Methods.

[CR23] Inghilleri M, Berardelli A, Cruccu G, Manfredi M (1993). Silent period evoked by transcranial magnetic stimulation of the human cortex and cervicomedullary junction. J Physiol.

[CR24] Kamen G, Knight CA (2004). Training-related adaptations in motor unit discharge rate in young and older adults. J Gerontol—Series A Biol Sci Med Sci.

[CR25] Kidgell DJ, Goodwill AM, Frazer AK, Daly RM (2013). Induction of cortical plasticity and improved motor performance following unilateral and bilateral transcranial direct current stimulation of the primary motor cortex. BMC Neurosci.

[CR26] Latella C, Teo W-P, Harris D, Major B, VanderWesthuizen D, Hendy A (2017). Effects of acute resistance training modality on corticospinal excitability, intra-cortical and neuromuscular responses. Eur J Appl Physiol.

[CR27] Manca A, Ginatempo F, Cabboi MP, Mercante B, Ortu E, Dragone D, De Natale ER, Dvir Z, Rothwell JC, Deriu F (2016). No evidence of neural adaptations following chronic unilateral isometric training of the intrinsic muscles of the hand: a randomized controlled study. Eur J Appl Physiol.

[CR28] Manning CD, Bawa P (2011). Heteronymous reflex connections in human upper limb muscles in response to stretch of forearm muscles. J Neurophysiol.

[CR29] Martin PG, Butler JE, Gandevia SC, Taylor JL (2008). Noninvasive stimulation of human corticospinal axons innervating leg muscles. J Neurophysiol.

[CR30] McDonnell MN, Orekhov Y, Ziemann U (2006). The role of GABAB receptors in intracortical inhibition in the human motor cortex. Exp Brain Res.

[CR31] McNeil CJ, Martin PG, Gandevia SC, Taylor JL (2009). The response to paired motor cortical stimuli is abolished at a spinal level during human muscle fatigue. J Physiol.

[CR32] McNeil CJ, Butler JE, Taylor JL, Gandevia SC (2013). Testing the excitability of human motoneurons. Front Hum Neurosci.

[CR33] Mills KR (1988). Excitatory and inhibitory effects on human spinal motoneurones from magnetic brain stimulation. Neurosci Lett.

[CR34] Nichols TR (2018). Distributed force feedback in the spinal cord and the regulation of limb mechanics. J Neurophysiol.

[CR35] Petersen NT, Taylor JL, Gandevia SC (2002). The effect of electrical stimulation of the corticospinal tract on motor units of the human biceps brachii. J Physiol.

[CR36] Pierrot-Deseilligny E, Burke D (2005). The circuitry of the human spinal cord.

[CR37] Piotrkiewicz M, Kudina L, Mierzejewska J, Jakubiec M, Hausmanowa-petrusewicz I (2007). Age-related change in duration of afterhyperpolarization of human motoneurones. J Physiol.

[CR38] Rossi S, Hallett M, Rossini PM, Pascual-Leone A, Avanzini G, Bestmann S, Berardelli A, Brewer C, Canli T, Cantello R, Chen R, Classen J, Demitrack M, Di Lazzaro V, Epstein CM, George MS, Fregni F, Ilmoniemi R, Jalinous R, Ziemann U (2009). Safety, ethical considerations, and application guidelines for the use of transcranial magnetic stimulation in clinical practice and research. Clin Neurophysiol.

[CR39] Ruotsalainen I, Ahtiainen JP, Kidgell DJ, Avela J (2014). Changes in corticospinal excitability during an acute bout of resistance exercise in the elbow flexors. Eur J Appl Physiol.

[CR40] Sidhu SK, Cresswell AG, Carroll TJ (2013). Corticospinal responses to sustained locomotor exercises: moving beyond single-joint studies of central fatigue. Sports Med.

[CR41] Škarabot J, Ansdell P, Brownstein CG, Thomas K, Howatson G, Goodall S, Durbaba R (2019). Electrical stimulation of human corticospinal axons at the level of the lumbar spinal segments. Eur J Neurosci.

[CR42] Škarabot J, Mesquita RNO, Brownstein CG, Ansdell P (2019). Myths and methodologies: how loud is the story told by the transcranial magnetic stimulation-evoked silent period?. Exp Physiol.

[CR43] Taylor JL (2006). Stimulation at the cervicomedullary junction in human subjects. J Electromyogr Kinesiol.

[CR44] Taylor JL, Butler JE, Allen GM, Gandevia SC (1996). Changes in motor cortical excitability during human muscle fatigue. J Physiol.

[CR45] Taylor JL, Butler JE, Gandevia SC (1999). Altered responses of human elbow flexors to peripheral-nerve and cortical stimulation during a sustained maximal voluntary contraction. Exp Brain Res.

[CR46] Taylor JL, Petersen NT, Butler B, Gandevia SC (2002). Interaction of transcranial magnetic stimulation and electrical transmastoid stimulation in human subjects. J Physiol.

[CR47] Taylor JL, Todd G, Gandevia SC (2006). Evidence for a supraspinal contribution to human muscle fatigue. Clin Exp Pharmacol Physiol.

[CR48] Triggs WJ, Cros D, Macdonell RAL, Chiappa KH, Fang J, Day BJ (1993). Cortical and spinal motor excitability during the transcranial magnetic stimulation silent period in humans. Brain Res.

[CR49] van Melick N, Meddeler BM, Hoogeboom TJ, Nijhuis-van der Sanden MWG, van Cingel REH (2017). How to determine leg dominance: the agreement between self-reported and observed performance in healthy adults. PLoS ONE.

[CR50] Vernillo G, Barbi C, Temesi J, Giuriato G, Giuseppe F, Martignon C, Schena F, Venturelli M (2022). Reliability of relaxation properties of knee-extensor muscles induced by transcranial magnetic stimulation. Neurosci Lett.

[CR51] Walker S, Blazevich AJ, Haff GG, Tufano JJ, Newton RU, Häkkinen K (2016). Greater strength gains after training with accentuated eccentric than traditional isoinertial loads in already strength-trained men. Front Physiol.

[CR52] Wilmink RJH, Nichols TR (2003). Distribution of heterogenic reflexes among the quadriceps and triceps surae muscles of the cat hind limb. J Neurophysiol.

[CR53] Yacyshyn AF, Woo EJ, Price MC, McNeil CJ (2016). Motoneuron responsiveness to corticospinal tract stimulation during the silent period induced by transcranial magnetic stimulation. Exp Brain Res.

[CR54] Zehr EP, Collins DF, Chua R (2001). Human interlimb reflexes evoked by electrical stimulation of cutaneous nerves innervating the hand and foot. Exp Brain Res.

[CR55] Ziemann U, Netz J, Szelényi A, Hömberg V (1993). Spinal and supraspinal mechanisms contribute to the silent period in the contracting soleus muscle after transcranial magnetic stimulation of human motor cortex. Neurosci Lett.

